# Exposure to marital conflict: Gender differences in internalizing and externalizing problems among children

**DOI:** 10.1371/journal.pone.0222021

**Published:** 2019-09-12

**Authors:** Rikuya Hosokawa, Toshiki Katsura

**Affiliations:** 1 School of Nursing, Nagoya City University, Nagoya, Japan; 2 Graduate School of Medicine, Kyoto University, Kyoto, Japan; Washington University in St. Louis, UNITED STATES

## Abstract

**Purpose:**

Marital conflict is integral to children’s psychosocial well-being. Extant research has shown that the effects of marital conflict on children are likely to vary by gender, indicating that gender plays a significant and complex role in the relationship between marital conflict and child adjustment. Focusing on gender, this study investigates the link between specific marital conflict tactics and children’s mental health symptoms in families in which the parents live together.

**Methods:**

This study gathered data from 799 children and their parents in Japan by means of a questionnaire focusing on marital conflict and child behavioral problems. Marital conflict (verbal aggression, physical aggression, stonewalling, avoidance-capitulation, child involvement, and cooperation) was assessed using a Conflict and Problem-Solving Scale. Children’s behavioral problems (externalizing and internalizing symptoms) were assessed using the Strengths and Difficulties Questionnaire.

**Results:**

The findings highlight the significant impact of specific interparental conflict on children’s behavioral problems, demonstrating that there are differences according to the child’s gender. More specifically, multivariate analyses targeting boys revealed that cooperation was significantly inversely associated with externalizing problems and internalizing problems, while avoidance-capitulation and verbal aggression were significantly positively associated with externalizing problems. In contrast, multivariate analyses targeting girls revealed that cooperation was significantly inversely associated with externalizing problems and internalizing problems, while avoidance-capitulation and stonewalling were significantly positively associated with internalizing problems.

**Conclusion:**

This study reveals that interparental conflict is associated with children’s behavioral problems. Constructive marital conflict was significantly inversely associated with externalizing and internalizing problems in both boys and girls. Meanwhile, destructive marital conflict (i.e., avoidance-capitulation and verbal aggression) was significantly positively associated with externalizing problems in boys and significantly positively associated with internalizing problems in girls. These findings contribute to the substantial literature demonstrating the relationship between family processes and the development of disruptive behavior disorders in children.

## Introduction

Marital conflict is unavoidable. Occurring in virtually all relationships, conflict is an inevitable part of family life. As such, children living in families witness marital conflict. It is clear that interparental relationships are integral to children’s psychosocial well-being [1,2]. Previous research has shown that the effects of marital conflict on children are likely to vary by gender, indicating that gender plays an important role in the relationship between marital conflict and child adjustment [3]. In other words, although both boys and girls are vulnerable to the effects of high marital conflict, gender appears to moderate potential outcomes [3]. For example, the threat associated with marital conflict may be more significant for boys, who tend to respond by externalizing problems. In contrast, girls evidence a tendency toward blaming themselves for parental conflict, thus developing internalizing problems. One study on adolescents found that parental hostility was associated with internalizing in both boys and girls, whereas externalizing was only significant in boys [4]. As such, gender may play a significant and complex role in the relationship between marital conflict and child adjustment. Thus, in order to understand how marital conflict impacts children, marital conflict tactics need to be explored with a focus on gender.

Numerous tactics can be adopted in the face of marital conflict. Where some deal with conflict in a positive and constructive manner, other tactics are negative and destructive. Extant studies have divided marital conflict tactics into two categories: namely, constructive and destructive. Constructive marital conflict tactics involve more positive strategies for ameliorating tension, including the open discussion of problems, resolving conflict calmly, and showing of affection during or after the conflict [5–7]. The use of constructive tactics elicits positive emotional reactions from children, such as secure attachment, better problem-solving skills, and emotional stability [8]. Constructive marital conflict may benefit positive child outcomes by teaching them problem-solving skills and effective ways of communicating, ultimately fostering more positive social relations. Positive outcomes include lower levels of internalizing (e.g., anxiety, depression, and withdrawal) and externalizing symptoms (e.g., aggression, delinquency, and conduct disorders), and higher levels of social skills, prosocial behavior, and emotion regulation [9,10].

In contrast, destructive marital conflict involves more negative conflict resolution tactics, including aggressive and threatening behavior, arguing frequently, and leaving issues unresolved [5,6]. Such destructive tactics may make children more vulnerable to developing adjustment problems, eliciting negative emotional and behavioral reactions such as aggression, conduct disorders, anxiety, and depression [5–7,11]. Exposure to destructive conflict is associated with increased symptoms of psychological distress among children [12,13]. Involving an implied threat to the emotional security of children, destructive marital conflict is associated with negative child outcomes including higher levels of internalizing and externalizing symptoms, and lower levels of social skills, prosocial behavior, and emotion regulation [14–17].

Accordingly, the presence of marital conflict and the way in which conflict is approached influence the environment in which the children learn and grow. Children are also likely to experience the indirect effects of marital conflict because it impacts parenting behaviors—the effects “spilling over” from the marital subsystem to the parent-child subsystem [18–20]. The association between marital conflict and children’s behavioral and emotional difficulties is well documented, with marital conflict found to be associated with a wide range of both externalizing and internalizing outcomes in children. Efforts to understand the association between marital conflict and child development have increasingly focused on how parents express and approach conflict in their relationship.

In approaching marital conflict, scholars differentiate between the ways in which parents interact and handle interpersonal conflicts on the one hand, and the behaviors and words exchanged during disagreements on the other [21]. Scholars have measured destructive marital conflict by the frequency of minor and major conflicts, the severity or degree of conflict, and conflict tactics. Destructive marital conflict contains various elements of avoidance, involving the child or children in the conflict, stonewalling, verbal aggression, and physical aggression [22–24]. As noted, conflict tactics influence the psychological well-being and development of children: children who live in families characterized by high levels of specific forms of interparental conflict are more likely to develop adjustment problems. Although extant studies have focused on destructive marital conflict through combined scale (e.g., stonewalling, verbal aggression, and physical aggression), these studies do not estimate the impact of specific conflict tactics on child outcomes. As such, it is necessary to explore specific conflict tactics in order to understand how marital conflict impacts children. This is particularly necessary because despite the likelihood of the effects of marital conflict on children varying according to gender, few studies have examined the influence of gender on the association between specific conflict tactics and child adjustment. Thus, in order to understand how marital conflict impacts children, specific conflict tactics need to be explored with a focus on gender. Further research is necessary to clarify the link between specific conflict tactics and gender—a line of enquiry that will aid in the provision of preventative measures at an early stage. This study addresses this gap.

Focusing on gender, this study investigates the link between specific interparental conflicts and children’s mental health symptoms in families in which the parents live together. More specifically, this study hypothesizes that specific interparental conflict tactics—namely, cooperation, avoidance-capitulation, stonewalling, verbal aggression, physical aggression, and child involvement—are uniquely associated with children’s internalizing and externalizing problems. Previous studies have not conclusively shown whether specific interparental conflicts impact girls and boys differently. Addressing this gap, this study investigates the moderating effect of gender on the relationship between specific interparental conflict and children’s internalizing and externalizing problems.

## Methods

### Subjects

This study forms part of a larger longitudinal study examining the effects of the child-rearing environment on the social development and adjustment of children. In 2014, we requested permission from all facilities in Nagoya city in Aichi prefecture, a major urban area in Japan, and conducted our survey at facilities where permission was obtained. As one of Japan’s leading cities, Nagoya city provides a representative sample of Japan. Subjects were recruited from 52 kindergartens and 78 nursery schools. To recruit subjects, self-reported questionnaires were distributed to all parents of five-year-old preschool children enrolled in the kindergartens and nursery schools. The parents of children provided written informed consent and agreed to participate. We have conducted an annual survey as the children age, following their progress from preschool to junior high school. The baseline was created via the first wave in 2014, during which we obtained the address of subjects. Each year, we mailed questionnaires to the parents of children (second wave in 2015, third wave in 2016). This study uses the data from the third wave survey.

The research for this study was conducted in 2016. Data were collected via self-administered questionnaires, which respondents completed manually using pen and paper. This study distributed one questionnaire per child, with the expectation that parents would answer jointly. Questionnaires were mailed to the parents of children, who completed the questionnaires and returned them to the researcher by mail. Self-report questionnaires were provided to subjects deemed eligible for the follow-up survey: the parents of seven-year-old children (*N* = 1,643) who were in the second grade of elementary school (child subjects were aged five during the first wave, and seven in the third wave). Of the 1,643 questionnaires disseminated, 935 completed questionnaires were returned. In order to accurately clarify the association between interparental relationships and child adjustment, this study excludes the following from its analysis: (1) children from single-parent families, (2) children diagnosed with developmental problems, and (3) children whose parents did not return complete questionnaires. To examine the impact of the specific interparental conflict tactics on children’s internalizing and externalizing problems as closely as possible, we excluded children diagnosed with developmental problems. We defined parents as mothers and fathers; while they did not need to be biologically related to the target child, they did need to reside with the child. Of the 935 children whose parents completed the questionnaire, 799 (85.5%) met the inclusion criteria. A sample size for analysis was not set because this study forms part of a larger longitudinal study. Nonetheless, the obtained sample size (*n* = 799) was large enough to detect linear regressions to assess associations between the interparental relationship and child outcomes [25].

Subject characteristics are shown in Table 1. The average age of the subjects was 8.02 years (*SD* = 0.36). In regard to gender, 51.8% of the subjects were boys (*n* = 414) and 48.2% girls (*n* = 385). The median annual household income was between JPY 5,000,000 and 5,999,999. Regarding subject mothers, the median age was 39.77 (*SD* = 4.42). In terms of their level of education, 1.9% had compulsory education, 20.2% had an upper secondary school diploma, 39.9% had up to four years of college/university education, and 38% had at least four years at college/university or higher degrees. In regard to subject fathers, the median aged was 41.87 (*SD* = 5.46). In terms of their level of education, 4.3% had compulsory education, 23.4% held an upper secondary school diploma, 14% had up to four years of college/university education, and 58.3% had at least four years at college/university or higher degrees. In terms of socioeconomic status, subjects’ socioeconomic status was similar to that typical of other Japanese individuals of a similar generation [26,27].

**Table 1:** Subject characteristics and emotional/behavioral problems.

	Boys (*N* = 414)								Girls (*N* = 385)							
			Externalizing Problems			Internalizing Problems					Externalizing Problems			Internalizing Problems		
	*N*	*%*	*Mean*	*SD*	*p*	*Mean*	*SD*	*p*	*N*	*%*	*Mean*	*SD*	*p*	*Mean*	*SD*	*p*
Annual Household Income (in JPY millions)																
< 3	25	6.1	6.40	3.09	0.015	4.40	2.73	0.031	22	5.9	5.57	3.73	0.002	3.52	2.80	0.321
3–5	189	46.1	6.26	3.86		3.39	2.83		159	42.3	4.53	3.33		3.46	2.54	
6–8	120	29.3	5.83	3.34		3.38	2.96		123	32.7	4.43	2.59		3.20	2.72	
≥ 9	76	18.5	4.73	2.78		2.88	2.56		72	19.1	3.41	2.33		2.87	2.17	
Maternal Education Level																
Compulsory education	11	2.7	7.46	4.63	0.038	4.15	3.02	0.154	4	1.0	6.67	2.50	0.003	4.67	2.58	0.096
Upper secondary school	86	20.9	6.52	3.63		3.58	2.95		74	19.4	4.93	3.34		3.78	2.97	
Up to four years at college/university	169	41.1	5.65	3.30		3.45	2.93		147	38.6	4.54	2.92		3.34	2.60	
More than four years at college/university	145	35.3	5.46	3.50		2.92	2.55		156	40.9	3.75	2.65		3.00	2.47	
Paternal Education Level																
Compulsory education	17	4.1	6.83	3.05	<0.001	6.64	1.62	0.422	17	4.5	6.72	3.77	<0.001	3.79	2.48	0.236
Upper secondary school	98	23.9	6.70	3.57		3.54	3.09		87	22.8	4.78	2.82		3.72	2.71	
Up to four years at college/university	70	17.1	6.78	3.65		3.06	2.69		41	10.8	4.60	2.65		3.74	3.04	
More than four years at college/university	225	54.9	5.10	3.34		3.29	2.84		236	61.9	3.90	2.85		3.14	2.55	

Abbreviations: Standard Deviation (SD), p-value (p).

### Ethical statement

Children’s parents were informed of the study’s objectives and procedures and made aware that they were not obligated to participate in the baseline survey. Parents provided written informed consent on behalf of their children prior to participating in this research. Ethical approval for this study was obtained from the Kyoto University Ethics Committee (E2322).

### Measures

Data were collected via questionnaires with several different scales covering a wide range of issues concerning the child-rearing environment and child developmental outcomes. However, this study only uses measures pertaining to interparental relationships and children’s psychosocial difficulties.

#### Interparental conflict

This study utilizes the Conflict and Problem-Solving Scale (CPS), a self-report questionnaire measuring the characteristics of the specific marital conflict strategies used by respondents [24]. The CPS is a 44-item questionnaire that uses a four-point Likert scale ranging from never (0) to often (3) to measure various aspects of marital conflict. The CPS comprises four conflict dimensions—frequency, severity, resolution, and efficacy—and six conflict strategy subscales: cooperation, avoidance-capitulation, stonewalling, verbal aggression, physical aggression, and child involvement. Cooperation involves the use of reasoning, problem-solving, and cooperation. Avoidance-capitulation involves attempts to ignore or escape arguments, while stonewalling refers to impasses in conflict characterized by unresolved hostility, distress, and disengagement. Verbal aggression pertains to the use of verbally hostile conflict tactics, while physical aggression involves the use of physical violence in interparental conflict. Finally, child involvement concerns the direct or indirect involvement of the child in the parents’ conflict. These scales have adequate internal consistency [24]. To address cross-cultural translation issues in this study, we back translated the items and checked their reliability according to the methodology recommended by Acquadro et al. [28]. In the sample used in this study, internal consistency coefficients range from 0.76 to 0.88 (Table 2).

**Table 2:** Descriptive statistics for the study variables (*N* = 799).

Description	Range	Mean	SD	α
Marital conflict: Conflict and Problem-Solving Scales (CPS)				
Cooperation	0–18	14.42	3.40	0.86
Avoidance-Capitulation	0–30	14.73	5.50	0.80
Stonewalling	0–21	3.25	3.33	0.77
Verbal Aggression	0–24	8.99	5.14	0.88
Physical Aggression	0–21	0.87	1.69	0.76
Child Involvement	0–15	4.34	3.11	0.77
Child’s Emotional/Behavioral Problems: Strengths and Difficulties Questionnaire (SDQ)				
Externalizing problems	0–20	5.12	3.32	0.70
Internalizing problems	0–20	3.32	2.73	0.74

Abbreviations: Standard Deviation (SD), Cronbach's Alpha (α).

#### Externalizing and internalizing problems in children

This study uses the Strengths and Difficulties Questionnaire (SDQ) to measure mental health problems in the child subjects, specifically externalizing and internalizing problems [29]. The SDQ is a 25-item questionnaire that uses a three-point Likert scale ranging from not true (0) to certainly true (2) to measure externalizing and internalizing behaviors. In the SDQ, the difficult behavior score is calculated as the sum of the scores obtained for the following subscale: emotional symptoms, conduct problems, hyperactivity, and peer problems.

Following the recommendation of Goodman et al. [30], this study combined the conduct problems and hyperactivity-inattention subscales into an externalizing problem scale, and the peer problems and emotional problems subscales into an internalizing problems scale. These scales have adequate internal consistency [29–31]. Additionally, the SDQ was cross-culturally validated for the Japanese context [31]. In this study, internal consistency coefficients were 0.70 for the externalizing problems scale, and 0.74 for the internalizing problems scale (Table 2).

#### Demographic covariates

Parents provided their demographic information, including their child’s sex, annual household income, maternal education level, and paternal education level. Annual household income was reported in Japanese yen (JPY). Regarding parental education, both parents were asked to report their education in years, as well as the highest level of education completed.

### Data analysis

First, the relationships between subject characteristics and children’s behavioral problems were analyzed via one-way analysis of variance (ANOVA), as shown in Table 1. Second, associations between the interparental relationship and child outcomes were assessed using multiple linear regression with CPS scores (cooperation, avoidance-capitulation, stonewalling, verbal aggression, physical aggression, and child involvement) as predictors and SDQ scores (externalizing problems and internalizing problems) as outcomes. The regression analyses were stratified by sex.

Regression analyses were organized as follows. In Model 1, each predictor was entered individually to assess its univariate association with each outcome (cooperation, avoidance-capitulation, stonewalling, verbal aggression, physical aggression, and child involvement). In Model 2, all predictors (cooperation, avoidance-capitulation, stonewalling, verbal aggression, physical aggression, and child involvement) were entered simultaneously. As several subject characteristics were significantly associated with behavioral problems in the analyses (see Table 1), we included the factors as covariates in each analysis (see Tables 3 and 4). Multicollinearity was assessed using variance inflation factor (VIF), and multicollinearity was considered when the VIF value exceeded 2. The VIF value was <2 indicating no multicollinearity among the predictors, and no problems with multicollinearity were found. All analyses were conducted using IBM SPSS Statistics 23.0.

**Table 3:** The link between interparental conflict and children’s externalizing problems.

	Model 1					Model 2				
	*B*	*SE*	β	*p*	*Adjusted R*^*2*^	*B*	*SE*	β	*p*	*Adjusted R*^*2*^
Boys										
Cooperation	-0.159	0.050	-0.156	0.002	0.065	-0.118	0.051	-0.117	0.022	0.150
Avoidance-Capitulation	0.114	0.031	0.178	<0.001	0.073	0.110	0.032	0.171	0.001	
Stonewalling	0.217	0.050	0.209	<0.001	0.086	0.011	0.068	0.010	0.876	
Verbal Aggression	0.141	0.033	0.211	<0.001	0.090	0.111	0.045	0.164	0.014	
Physical Aggression	0.351	0.104	0.164	0.001	0.068	0.169	0.136	0.071	0.214	
Child Involvement	0.193	0.055	0.170	0.001	0.069	0.033	0.070	0.029	0.634	
Girls										
Cooperation	-0.113	0.043	-0.132	0.010	0.070	-0.090	0.046	-0.106	0.048	0.106
Avoidance-Capitulation	0.046	0.027	0.087	0.092	0.059	0.025	0.028	0.047	0.382	
Stonewalling	0.128	0.044	0.150	0.004	0.072	0.056	0.061	0.066	0.362	
Verbal Aggression	0.051	0.028	0.091	0.073	0.065	0.017	0.041	0.031	0.671	
Physical Aggression	0.172	0.078	0.114	0.028	0.062	0.084	0.095	0.054	0.377	
Child Involvement	0.071	0.048	0.075	0.139	0.057	0.011	0.063	0.012	0.859	

*Note*. Model 1: Each predictor was entered individually to assess its univariate association with each outcome (Cooperation, Avoidance-Capitulation, Stonewalling, Verbal Aggression, Physical Aggression, and Child Involvement). Model 2: All predictors (Cooperation, Avoidance-Capitulation, Stonewalling, Verbal Aggression, Physical Aggression, and Child Involvement) were entered simultaneously.

Abbreviations: Unstandardized coefficient (B), Standard Error (SE), Standardized coefficient (β), p-value (p).

**Table 4:** The link between interparental conflict and children’s internalizing problems.

	Model 1					Model 2				
	*B*	*SE*	β	*p*	*Adjusted R*^*2*^	*B*	*SE*	β	*p*	*Adjusted R*^*2*^
Boys										
Cooperation	-0.100	0.042	-0.119	0.019	0.028	-0.092	0.045	-0.110	0.040	0.105
Avoidance-Capitulation	0.051	0.026	0.098	0.053	0.025	0.035	0.028	0.065	0.216	
Stonewalling	0.069	0.043	0.081	0.111	0.022	-0.011	0.061	-0.013	0.857	
Verbal Aggression	0.033	0.028	0.060	0.234	0.020	0.042	0.040	0.077	0.290	
Physical Aggression	0.132	0.086	0.077	0.125	0.022	0.134	0.117	0.069	0.253	
Child Involvement	0.035	0.046	0.038	0.449	0.017	-0.052	0.060	-0.057	0.390	
Girls										
Cooperation	-0.098	0.041	-0.124	0.017	0.035	-0.077	0.031	-0.095	0.012	0.120
Avoidance-Capitulation	0.098	0.026	0.197	<0.001	0.060	0.081	0.026	0.165	0.002	
Stonewalling	0.191	0.040	0.247	<0.001	0.079	0.109	0.056	0.139	0.044	
Verbal Aggression	0.081	0.027	0.156	0.003	0.049	0.021	0.037	0.041	0.572	
Physical Aggression	0.193	0.073	0.139	0.008	0.038	0.107	0.087	0.074	0.221	
Child Involvement	0.125	0.045	0.143	0.006	0.040	0.005	0.058	0.005	0.937	

*Note*. Model 1: Each predictor was entered individually to assess its univariate association with each outcome (Cooperation, Avoidance-Capitulation, Stonewalling, Verbal Aggression, Physical Aggression, and Child Involvement). Model 2: All predictors (Cooperation, Avoidance-Capitulation, Stonewalling, Verbal Aggression, Physical Aggression, and Child Involvement) were entered simultaneously.

Abbreviations: Unstandardized coefficient (B), Standard Error (SE), Standardized coefficient (β), p-value (p).

## Results

### Subject characteristics and emotional/behavioral problems

Subjects’ demographic characteristics and the relationships between these characteristics and behavioral problems are shown in Table 1. In regard to boys, the externalizing and internalizing problem scores of boys in lower income households were significantly higher than those of boys in higher income households. More specifically, externalizing problem scores: < JPY 3 million, 6.40±3.09; JPY 3–5 million, 6.26±3.86; JPY 6–8 million, 5.83±3.34; ≥ JPY 9 million, 4.73±2.78; *p* = 0.015. Internalizing problem scores: < JPY 3 million, 4.40±2.73; JPY 3–5 million, 3.39±2.83; JPY 6–8 million, 3.38±2.96; ≥ JPY 9 million, 2.88±2.56; *p* = 0.031. In regard to maternal education level, externalizing problem scores were significantly higher among boys whose mothers had a lower education level. Externalizing problem scores: compulsory education, 7.46±4.63; upper secondary school, 6.52±3.63; up to four years at college/university, 5.65±3.30; more than four years at college/university, 5.46±3.50; *p* = 0.038. In regard to paternal education level, children’s externalizing problem scores were significantly higher among children whose fathers had a lower education level. Externalizing problem scores: compulsory education, 6.83±3.05; upper secondary school, 6.70±3.57; up to four years at college/university, 6.78±3.65; more than four years at college/university, 5.10±3.34; *p* < 0.001.

In contrast, externalizing problem scores were significantly higher among girls in lower income households than those of girls in higher income households. Externalizing problem scores: < JPY 3 million, 5.57±3.73; JPY 3–5 million, 4.53±3.33; JPY 6–8 million, 4.43±2.59; ≥ JPY 9 million, 3.41±2.33; *p* = 0.002. In regard to maternal education level, externalizing problem scores were significantly higher among girls whose mothers had a lower education level. Externalizing problem scores: compulsory education, 6.67±2.50; upper secondary school, 4.93±3.34; up to four years at college/university, 4.54±2.92; more than four years at college/university, 3.75±2.65; *p* = 0.003. In regard to paternal education level, externalizing problem scores were significantly higher among girls whose fathers had a lower education level. Externalizing problem scores: compulsory education, 6.72±3.77; upper secondary school, 4.78±2.82; up to four years at college/university, 4.60±2.65; more than four years at college/university, 3.90±2.85; *p* < 0.001.

### The link between interparental conflict and children’s emotional/behavioral problems

The associations between interparental conflict and children’s outcomes were assessed using multiple linear regression with CPS and SDQ scores as outcomes (see Tables 3 and 4). First, in regard to externalizing problems, Table 3 shows the results of the multivariate analysis of the relationships between interparental conflict and emotional/behavioral problems. Regarding boys, in Model 1—which included individual predictors—all predictors (i.e., cooperation, avoidance-capitulation, stonewalling, verbal aggression, physical aggression, and child involvement) were significantly associated with externalizing problems. In Model 2—which contained all predictors—cooperation was significantly inversely associated with externalizing problems (β = -0.117, *p* = 0.022), while avoidance-capitulation (β = 0.171, *p* = 0.001) and verbal aggression (β = 0.164, *p* = 0.014) were significantly positively associated with externalizing problems. In contrast, only some of the predictors—namely, cooperation, stonewalling, and physical aggression—in Model 1 were significantly associated with externalizing problems in girls. In Model 2, cooperation was significantly inversely associated with externalizing problems (β = -0.106, *p* = 0.048).

Second, in regard to internalizing problems, Table 4 shows the results of the multivariate analysis of the relationships between interparental conflict and emotional/behavioral problems. Regarding boys, only one predictor—cooperation—was significantly associated with internalizing problems in Model 1. In Model 2, cooperation was significantly inversely associated with internalizing problems (β = -0.110, *p* = 0.040). Analyses of data pertaining to girls produced different results. In Model 1, all predictors (cooperation, avoidance-capitulation, stonewalling, verbal aggression, physical aggression, and child involvement) were significantly associated with internalizing problems. In Model 2, cooperation was significantly inversely associated with internalizing problems (β = -0.095, *p* = 0.012), while avoidance-capitulation (β = 0.165, *p* = 0.002) and stonewalling (β = 0.139, *p* = 0.044) were significantly positively associated with internalizing problems.

## Discussion

The purpose of this study was to examine the relationship between specific interparental conflict tactics and children’s internalizing and externalizing problems with a focus on gender. The findings of this study highlight the significant impact of specific interparental conflict on children’s behavioral problems, demonstrating that these vary according to the gender of the child. More specifically, multivariate analysis targeting boys revealed that cooperation was significantly inversely associated with both externalizing and internalizing problems, while avoidance-capitulation and verbal aggression were significantly positively associated with externalizing problems. In contrast, multivariate analysis targeting girls revealed that cooperation was significantly inversely associated with both externalizing and internalizing problems, while avoidance-capitulation and stonewalling were significantly positively associated with internalizing problems. In short, this study found that constructive marital conflict was inversely related to externalizing and internalizing problems in both boys and girls. However, boys tend to respond to specific destructive marital conflict by manifesting adjustment problems in terms of externalizing behavior, whereas girls tend to respond with internalizing behavior. These findings are consistent with those of previous studies [3].

It is worth noting that destructive marital conflict associated with behavioral problems not only includes active conflict such as verbal aggression, but silent conflict such as avoidance-capitulation and stonewalling. Numerous studies have focused on active conflict tactics—such as verbal hostility and physical aggression—as forms of destructive conflict or understood destructive conflict as including both active and silent conflict tactics (e.g., stonewalling and withdrawal or avoidance). Such studies have suggested that these types of destructive marital conflict tactics negatively influence child outcomes [5–7,17]. However, this study found that active conflict tactics and silent conflict tactics—such as avoidance-capitulation and stonewalling—were independently significantly associated with behavioral problems. Avoidance-capitulation involves passive strategies that minimize confrontation and maintain harmony at all costs. Stonewalling is the passive-aggressive withdrawal from active conflict through behavior such as sulking and giving one’s partner the “silent treatment.” These strategies are relatively ineffective because they do not allow the underlying source of conflict to be resolved [24]. In contrast, cooperation is an effective conflict strategy that involves an attempt to “meet halfway,” ensuring that the needs of both individuals are met. The spillover hypothesis suggests that the negativity or positivity experienced in the interparental relationship may influence or transfer to the parent-child relationship [32,33]. As such, silent conflict tactics may be transferred to the parent-child relationship. A lack of parental emotional or physical availability interferes with the child’s ability to form secure attachments and receive emotional support [34,35]. Defined as the omission of sufficient care to meet a child’s basic needs, child neglect is a major risk factor in psychopathology, including the development of internalizing and externalizing problems [36,37]. Poor parent-child relationships put children at risk of pervasive and cumulative consequences across their lifespan.

In this study, destructive marital conflict tactics—specifically, avoidance-capitulation and verbal aggression—were significantly positively associated with externalizing problems in boys. In contrast, destructive marital conflict tactics—specifically, avoidance-capitulation and stonewalling—were significantly positively associated with internalizing problems in girls. Several mechanisms are likely to be different between girls and boys in terms of the relationship between marital conflict and the risk of developing emotional and behavioral problems. Although both boys and girls are vulnerable to the effects of marital conflict, gender appears to moderate the potential outcomes and processes by which this relationship occurs [3]. In other words, the reaction of children to marital conflict tends to differ according to gender. As noted, prior research has suggested that boys tend to respond to marital conflict by manifesting adjustment problems in terms of externalizing behavior, whereas girls tend to respond with internalizing behavior [3]. In this study, destructive marital conflict tactics were significantly positively associated with externalizing problems in boys, while destructive marital conflict tactics were significantly positively associated with internalizing problems in girls. The literature suggests some explanations for this.

Gender differences in the socialization process may affect how boys and girls form appraisals and coping behaviors [38]. Boys may be more reactive to stress and better observers of marital conflict than girls [3]. Although there is evidence that boys and girls are similarly exposed to their parents’ conflicts, some studies suggest that parents may feel boys are more resilient than girls and consequently feel less obligated to shield them from conflict [39]. In this study, while boys and girls are similarly exposed to their parents’ conflicts, boys tend to be exposed to more strict parenting attitudes than girls (see S1 Table). Boys have also been found to be more likely to propose task-oriented interventions for parental conflict than girls [40]. Providing a perspective on how parents’ relationships influence the relationship experiences of children in adulthood, social learning theory suggests that children learn social behavior, in part, from observing their caregivers’ social interactions and relationships [41]. Indeed, boys exposed to marital conflict may be more likely to replicate parental anger [42]. Boys may feel especially threatened by marital conflict and react with aggressive behavior, often responding to adult anger with aggression and anger. As such, appraisals of coping efficacy and the threat posed by marital conflict may be more crucial for boys because boys tend to respond with adjustment and behavioral problems.

In contrast to boys, girls exposed to marital conflict tend to evidence more internalizing symptoms. Girls are more likely to react to marital conflict with fear [40] and have been found to exhibit distress in the face of adult anger [43]. It may be that the change from a secure to insecure environment and the resultant loss of emotional security as a result of marital conflict feels especially threatening to girls [3]. They tend to respond to stress with self-blaming behavior and other cognitions associated with internalizing symptoms. As such, evidencing a tendency toward self-blame for parental conflict, girls tend to develop internalizing problems.

### Limitations

This study has several limitations. First, the study design was a cross-sectional study. The direction of effect between marital conflict and children’s behavioral problems is usually assumed to be that marital conflict impacts upon children’s behavior. However, some studies have reported bidirectional relations between interparental conflict and children’s behavioral problems, noting that a child’s problematic behavior is likely to interact with interparental conflict in predicting developmental outcomes [44]. There are also bidirectional relations between a child’s irritable and defiant temperament and destructive marital conflict. For instance, temperamental precursors, physiological responsivity to stress, and other child characteristics have been shown to have a reciprocal relationship with the environment to influence the development of psychopathology [45,46].

Second, in this study, the CPS and SDQ were only completed by caregivers, likely introducing reporting bias. Regarding the CPS, questionnaire data are a less robust means of quantifying parental discipline than direct observation. Consequently, the completion of a single observation opinion-based questionnaire can be considered a crude and potentially inadequate means of obtaining data about marital conflict. Future research should explore marital conflict strategies from the perspectives of both children and parents. Regarding the SDQ, reports from other parties—such as teachers—may help in evaluating the dynamics more accurately. As such, future research should combine teacher and caregiver SDQ scores.

Third, as the purpose of this study was to examine the relationship between specific interparental conflict tactics and children’s internalizing and externalizing problems, we did not include parenting styles in the analysis. Future studies should look at whether specific marital conflict resolution mechanisms match parenting styles in order to test this spillover hypothesis.

Fourth, the impact of certain marital conflicts may be influenced by cultural differences. For instance, a previous study verifying differences in how marital conflict is operationalized in Japan versus the United States suggests that the degree of conjugal verbal and physical aggression was exceedingly high in American spouses followed by Japanese couples [47]. This finding may reflect the nature of culture in each society, specifically an expressive American culture and reserved Japanese culture. As this study considers Japanese subjects, it may underestimate the impact of verbal and physical aggression on children in comparison to other countries.

Fifth, this study’s definition of ‘parents’ did not require that they be biologically related to the subject. The mixed sample—comprising both biologically related and unrelated parents—may have influenced the link between specific marital conflict tactics and children’s mental health symptoms. As such, further study should consider biological relatedness.

Finally, this study did not use a genetically informed design, preventing the exploration of genetics or biological factors. This may be worth pursuing in future research. For instance, marital stress is associated with elevations in child cortisol levels and the later emergence of behavioral developmental symptoms [48]. It is also important to consider child temperament as it may moderate the association of family adversities with child outcomes or may act in reciprocal relationships with these factors. As such, further study should take genetic factors into account.

## Conclusion

The findings of this study demonstrate that interparental conflict is associated with children’s behavioral problems. This study found that constructive marital conflict tactics were significantly inversely associated with externalizing and internalizing problems in both boys and girls. Destructive marital conflict tactics—specifically avoidance-capitulation and verbal aggression—were significantly positively associated with the development of externalizing problems in boys. In contrast, destructive marital conflict tactics—specifically avoidance-capitulation and stonewalling—were significantly positively associated with the development of internalizing problems in girls. Thus, it is important to distinguish specific marital conflict tactics and consider the child’s gender when examining the relationship between marital or interparental conflict and its impact on a child’s psychological well-being. Furthermore, active conflict tactics (such as verbal aggression) and silent conflict tactics (such as avoidance-capitulation and stonewalling) were independently significantly associated with externalizing or internalizing behavioral problems. The associations between child symptoms and exposure to specific marital conflict highlight the need for effective prevention and intervention strategies. Moreover, these findings add to the substantial literature documenting the relationship between family processes and the development of disruptive behavior disorders in children. It is vital that further efforts to understand the association between marital conflict and child development focus on how parents express and manage conflicts in their relationship.

## Supporting information

S1 Table Exposure to marital conflict and parenting practices by child gender. (DOCX)
