# Supplementary material for: Exposure to marital conflict: Gender differences in internalizing and externalizing problems among children
Source: PLoS One. 2019 Sep 12;14(9):e0222021. doi: 10.1371/journal.pone.0222021 (PMC6742467; doi:10.1371/journal.pone.0222021)
Supplement: S1 Table — (DOCX) [file pone.0222021.s001.docx]

| **S1 Table. Exposure to marital conflict and parenting practices by child gender** | | | | | |
| --- | --- | --- | --- | --- | --- |
|  | Boys (*N* = 414) | | Girls (*N* = 385) | |  |
|  | *Mean* | *SD* | *Mean* | *SD* | *p* |
| Marital conflict: Conflict and Problem-Solving Scales (CPS) |  |  |  |  |  |
| Cooperation | 14.52 | 3.44 | 14.32 | 3.35 | 0.394 |
| Avoidance-Capitulation | 14.46 | 5.52 | 15.03 | 5.46 | 0.149 |
| Stonewalling | 3.22 | 3.35 | 3.29 | 3.31 | 0.754 |
| Verbal Aggression | 9.06 | 5.21 | 8.91 | 5.07 | 0.681 |
| Physical Aggression | 0.91 | 1.63 | 0.83 | 1.75 | 0.512 |
| Child Involvement | 4.30 | 3.15 | 4.38 | 3.07 | 0.700 |
| Parenting practice: Alabama Parenting Questionnaire (APQ) |  |  |  |  |  |
| Poor monitoring/supervision | 13.94 | 3.31 | 13.79 | 3.59 | 0.536 |
| Inconsistent discipline | 14.92 | 3.41 | 14.67 | 3.45 | 0.313 |
| Corporal punishment | 6.95 | 2.00 | 6.19 | 2.06 | <0.001 |
| Positive parenting | 21.33 | 3.19 | 20.96 | 3.51 | 0.126 |
| Involvement | 38.51 | 5.18 | 38.70 | 5.34 | 0.599 |
| Abbreviations: Standard Deviation (SD), p-value (p). |  |  |  |  |  |
